# The American Medical Association and Medical Education

**Published:** 1857-08

**Authors:** 


### THE AMERICAN MEDICAL ASSOCIATION AND MEDICAL EDUCATION.

Some of our editorial friends seem to be very much "*exercised*" on account of the introduction of certain resolutions in reference to medical education, into the American Medical Association, by our friend and colleague, Dr. J. Boring. Now, while we do not know the man in this broad land who is more competent to defend any position he may choose to take than the gentleman alluded to, and therefore requires no assistance from us, yet we feel inclined to make a few remarks upon this subject.

The truth is, it requires no labored argument to show that the resolutions referred to, embody the *facts* as to the *powers*, and what should be the *objects* of the association, and what are indeed understood to be the great ends to be accomplished by all medical organizations, and in addition to this, these resolutions exhibit in the most faithful manner, the *facts* as to the result of the effort, upon the part of that body, to regulate medical education, and seek to suggest the course in reference to this "vexed question" which would be most likely to result in the accomplishment of the great ends *stated*, as coming legitimately within its powers.

In reference to the first point, we would enquire where the association derived the "*power* to control" medical education, and if it has such a prerogative, why has it not been exercised? The truth is, no such power exists, and in the very nature of things it is *impossible* that it could. We are not ignorant of the fact that one of the objects of the establishment of the association was the elevation of the standard of medical education; the gentleman who introduced the resolutions embodying "the truth, the whole truth, and nothing but the truth" upon this subject, was not ignorant of this, as he is not likely to be lacking in knowledge of any fact that may have the most remote connection with any subject which he touches, but we presume that he was equally well acquainted with the *notorious* fact that the association had been found to be utterly impotent to the accomplishment of this end, not possessing, as is acknowledged upon all hands, any *legislative power* over the profession at large, and not possessing the "*moral power*" to induce compliance with its *recommendations*, from the equally notorious fact, that the Association does not and cannot embody the *sentiments* and *will* of the Medical profession of the United States, from a number of causes, among which we may designate its *origin*, not springing from the profession, but a few self constituted (virtually) guardians of the interests of the profession, a majority of whom were doubtless actuated by high and commendable motives, but utterly incompetent to the task of regulating medical affairs in this country, because *those to be controlled were not represented*, and we hesitate not to say, without the fear of successful contradiction, that this Association has continued, up to the present day, radically defective in the essential principles which belong to *legislation* in a free and enlightened government, and with the same confidence we assert that it has been equally defective in those elements which are indispensably necessary to arm it with the *moral* power required for the *control* of the profession, or even reform in any *radical* particular, no powers having been delegated to it and the profession generally, never having been represented in it, or recognized the small portion constituting it, as the *rightful* exponent of the medical sentiment or will of this vast country.

It is to be distinctly understood at the same time, that we contend honestly for what we *know* to be the *truth* in this matter, that we believe this organization has accomplished a great deal for the science of medicine and for the elevation of the profession in many ways, and we would give most cheerfully our feeble aid to cherish and sustain it as an instrument for good, which it can only be, by keeping within the sphere of practicable things.

But of all the positions in reference to this subject, that which strikes us as the most singular, is the one which suggests the securing of charters for medical colleges from the Congress of the United States; surely the author of such a proposition must have forgotten that there were such things as *sovereign* and *independent* States in this Union, not only competent to control their own internal affairs, but possessing a *supreme* control, except so far as they have seen fit to *surrender* certain rights, and to *permit* national legislation upon certain matters, for the general good. We do not mean to be misunderstood; we are no advocates for the doctrine, "that State Legislatures can create members of the medical profession," such as ought at least to be recognized, by those who are devoted to the advancement of medical *science,* and yet we know, and every body knows, that no *power* in a political sense to legislate upon and to regulate medical matters resides any where else than in the *State Governments:* this fact is too palpable to admit of being made plainer, notwithstanding all the rant and stuff in certain quarters about "medical freemen," &c., &c., coming as it does most strangely from those who would concentrate all power in the Federal Government. No sir, if you have been ignorant of the fact heretofore, learn that there are "State rights" in medicine as well as in other matters, and that the "medical freemen" of this country will never yield the *right* of regulating their own affairs, by their own *State medical organizations,* as they will never yield these same *rights,* in a political sense, to a national government.

So far as the proposition, to establish a uniform system of medical education is concerned, we do not hesitate to admit that it would be best, if it were practicable, to make it the result of the *deliberation*, the *will* and the *wants* of the medical profession of the United States, that it is impossible to obtain this in so vast and diversified a country as ours is too plain to admit of question, and, under existing circumstances, will only be attempted by visionaries or for the advancement of selfish ends. As we have remarked upon a former occasion, we are ready to go into the advocacy of any practical, feasible and common sense extension of facilities for teaching or requisitions for the doctorate, but do not believe that any radical change in the present plan will be sustained either by the profession or those desiring to enter it, or that more uniformity of action upon the part of the colleges would be secured by any other which might be adopted. The true friends of the association and those who do not wish to lose its labor for good will let this subject rest or will at least attempt no compulsory measures, even though it be admitted that "those composing the body have the right to choose their associates," while those whose motto is "rule or ruin" will continue to agitate, and, should their counsels prevail, will soon see the end of which, we fear, they have already seen the beginning.

